# Infection, Inflammation and Healing in Zebrafish: Intestinal Inflammation

**DOI:** 10.1007/s40139-015-0079-x

**Published:** 2015-04-05

**Authors:** Lindsay Marjoram, Michel Bagnat

**Affiliations:** Department of Cell Biology, Duke University Medical Center, Durham, NC 27710, Tel: 919-684-4899, lindsay.marjoram@duke.edu; Department of Cell Biology, Duke University Medical Center, Durham, NC 27710, Tel: 919-681-9268 Fax: 919-684-5481, m.bagnat@cellbio.duke.edu

**Keywords:** intestinal inflammation, zebrafish, inflammatory bowel disease, zebrafish disease models

## Abstract

Inflammatory bowel diseases (IBD), which include Crohn’s disease and ulcerative colitis, contribute to significant morbidity and mortality globally. Despite an increase in incidence, IBD onset is still poorly understood. Mouse models of IBD recapitulate several aspects of human disease, but limited accessibility for live imaging and the lack of forward genetics highlight the need for new model systems for disease onset characterization. Zebrafish represent a powerful platform to model IBD using forward and reverse genetics, live imaging of transgenic lines and physiological assays. In this review, we address current models of IBD in zebrafish and newly developed reagents available for future studies.

## Introduction

Inflammatory bowel diseases (IBD), which include Crohn’s disease and ulcerative colitis, contribute to significant morbidity and mortality globally [1]. Although the incidence of IBD is increasing, disease onset is still poorly understood [1]. The prevailing view is that IBD onset arises due to the intersection of multiple factors including genetic susceptibility, intestinal microbiota, aberrant immune system activity and environmental agents [1]. Mouse models of IBD have been developed and recapitulate several aspects of human disease [2], but the limited accessibility for imaging studies and the lack of forward genetics signify the need to develop other model systems to characterize disease onset.

Zebrafish have emerged as a powerful platform for the study of human disease states such as cancer, neurodegenerative disease and heart disease [3–6]. Optical transparency during embryonic and larval stages allows for high-resolution imaging of internal organs such as the intestine in live animals during development. A fully sequenced genome and newly developed high-throughput sequencing approaches make zebrafish a robust system for forward genetic screens [7•]. In zebrafish forward genetic screens, mutant phenotypes of interest can be identified visually and characterized with the aid of fluorescent reporter lines. Newly developed genome editing technologies (*e.g*. CRISPRs) have been successfully implemented in zebrafish, allowing for reverse genetic and knock-in approaches as well [8••]. Zebrafish are also amenable to high-throughput chemical screens. In this review, we discuss the technical advantages of the zebrafish for studying intestinal inflammation and recent focused efforts to develop zebrafish IBD models.

## Zebrafish as a model to study intestinal inflammation

In the context of intestinal development and disease, zebrafish have an intestinal tract that is homologous to mammals. Although Paneth cells and Peyer’s patches have not yet been identified and zebrafish lack a submucosa, the intestinal architecture, cell types and organ function are largely the same [9, 10]. In recent years, several groups have begun to use zebrafish as a model to study intestinal inflammation and intestinal disease. Adult zebrafish can spontaneously develop intestinal neoplasias and mutant lines (e.g. *apc*−/+) have also been generated as models to study cancer [3] (reviewed in [11]). Recently, several groups have begun to employ the strengths of the zebrafish system to study intestinal inflammatory diseases such as IBD [12].

**Figure 1:**
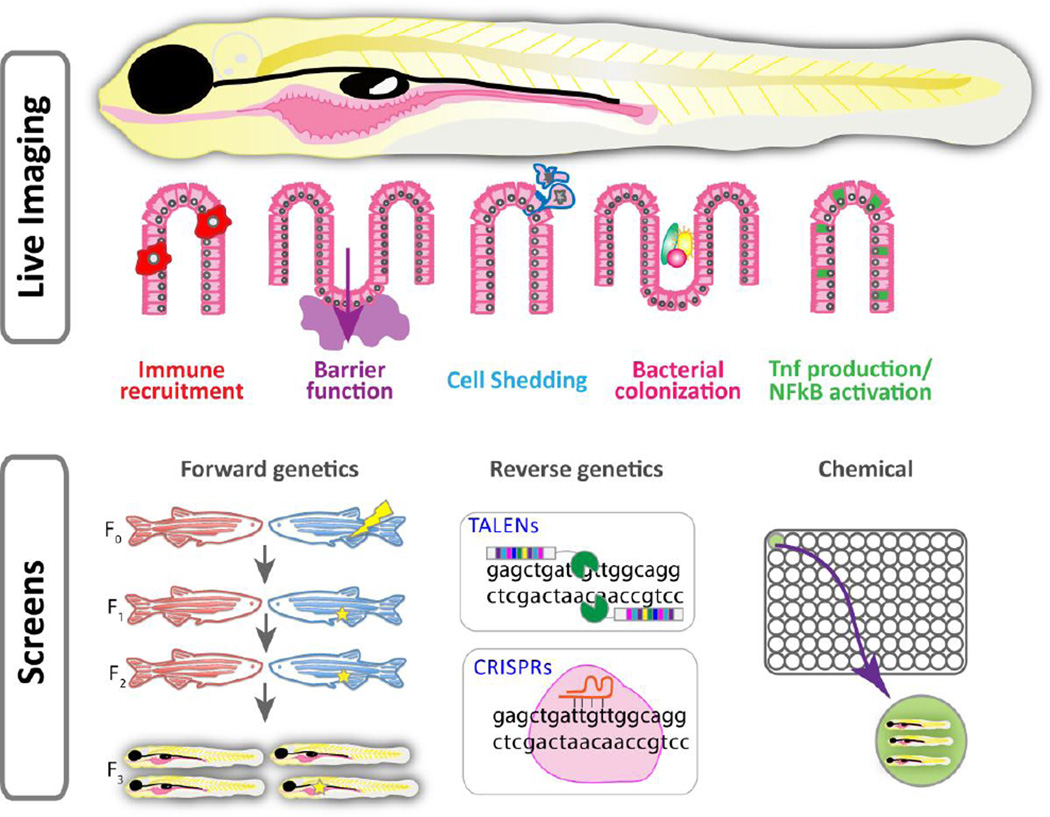
A zebrafish platform for the comprehensive study of IBD onset Live-imaging in zebrafish allows for the study of immune recruitment, barrier function, cell shedding, bacterial colonization and production of pro-inflammatory cytokines (top panel). Zebrafish are amenable to forward genetic screens, reverse genetics and library screens (bottom panel).

Although it is difficult to fully recapitulate IBD in any model organism due to the complex nature of the disease, there are several criteria that should and can be met in the zebrafish system to generate informative models (Figure 1, top panel). **1)** Zebrafish models of IBD should display altered intestinal morphology. In IBD, inflammation causes severe perturbations in the intestinal epithelium such that villi become blunted and epithelial erosion can occur, exposing the underlying tissues [13]. Moreover, there is increased apoptosis of IECs and accompanying defects in the barrier function of the epithelium [14]. Thus, a fish model of IBD should display alterations in epithelial morphology such as flattening of the intestinal folds, accelerated cell shedding and/or barrier defects. **2)** The immune system plays a vital role in IBD onset, and a zebrafish IBD model should show increased recruitment of immune cells to the intestine. However, because the fish adaptive immune system does not develop until 4–6 weeks of age [15], all larval IBD models will include only the innate immune system. **3)** There should also be increased expression of pro-inflammatory cytokines such as tumor necrosis factor (Tnfa), nuclear factor kappa-light-chain-enhancer of activated B cells (NF-κB), interleukin 1b (Il-1b) or matrix metallopeptidase 9 (mmp9). **4)** A fish IBD model may also be influenced by the intestinal microbiota such that germ-free derivation of the animals may alleviate or reduce intestinal inflammation.

## Chemical models of enterocolitis

Several chemical-based models of enterocolitis induction have been adapted from the murine system into both larval and adult fish. Although chemical induction of enterocolitis does not fully recapitulate all aspects of IBD, most chemical induction models perturb the intestinal epithelium either through damage or immune recruitment [14, 16]. Zebrafish are well suited to chemical induction of inflammation as large numbers of larvae can be analyzed at one time. Most chemical induction studies immerse larvae in a chemical over a short period of time (days), allowing the animals to swallow the chemical to create intestinal exposure. While chemical immersion is convenient, it can result in nonspecific extraintestinal defects [12]. This caveat may be circumvented by using a recently developed oral microgavage approach (detailed below) [17•], which allows for chemicals to be delivered directly to the intestine.

### Trinitrobenzene sulfonic acid (TNBS) and oxazolone

TNBS, a haptenizing agent, is thought to render colonic proteins immunogenic to the host organism and has been a standard tool for induction of intestinal inflammation in mice [2]. Culturing zebrafish larvae in TNBS from 3 days post-fertilization (dpf) through 8 dpf has been shown to elicit a broad but disparate range of intestinal phenotypes, perhaps due to the limitations of the immersion approach. While Fleming *et al*. and He *et al*. documented changes in intestinal morphology and increased numbers of goblet cells after exposure to TNBS [18, 19], Oehlers and colleagues did not observe severe intestinal morphological changes but did observe shortening of the mid-intestine (segment II) and significant intestinal recruitment of neutrophils [20]. Despite the morphological differences, all three studies report an increase in tumor necrosis factor (*tnfa*) expression [18–20]. TNBS treatment also resulted in increased *hsp70* and *hspa4* expression [21]. Addition of broad-spectrum antibiotics improved survival in TNBS-treated fish, suggesting that microbiota play a role in the inflammatory process [20]. Treatment with the clinically relevant agents prednisolone and 5-ASA rescued the inflammatory phenotype [18, 20]. Adult zebrafish studies have also been performed with TNBS. Intrarectal administration of TNBS disrupted intestinal morphology and resulted in increased recruitment of neutrophils but did not affect goblet cell number [22]. Melanin-concentrating hormone, a neuropeptide implicated in IBD pathogenesis, was also increased in the intestine after TNBS treatment. Increased apoptosis and cell shedding were not documented in these studies and barrier function was not examined.

Oxazolone, another type of hapten, has also been used to induce experimental colitis. Intrarectal administration of oxazolone in adult fish led to enterocolitis in fish raised in low flow tanks but not in continuous-flow tanks, a factor the authors attribute to the lower bacterial burden in continuous-flow fish [23]. Oxazolone-exposed fish develop significant alterations in their intestinal morphology, with blunted epithelial folds, loss of goblet cells, increased pro-inflammatory gene expression and infiltration of eosinophils [23]. Interestingly, vancomycin-induced alterations in the intestinal microbial population decreased the severity of enterocolitis induction while alterations induced by colistin sulfate were not effective [23]. Barrier function was not assayed.

### Dextran sodium sulfate (DSS)

DSS is a detergent thought to injure the intestinal epithelium, thereby causing a barrier defect. DSS administration to larval zebrafish produced similar outcomes to TNBS treatment, with intestinal recruitment of leukocytes and elevated pro-inflammatory gene expression, though it did not cause elevated intestinal apoptosis [24]. Different from TNBS-induced enterocolitis, however, is the presence of a mucosecretory phenotype with excess mucus production in the intestinal bulb. While inflammation could be reversed with addition of dexamethasone, the mucosecretory phenotype was separate, abolished by the addition of exogenous retinoic acid and protective against further insult from TNBS [24].

### Glafenine

Zebrafish bathed in the non-steroidal anti-inflammatory drug (NSAID) glafenine show a pronounced intestinal cell shedding and apoptosis phenotype that occurred hours after exposure but, surprisingly, did not lead to a barrier defect [25]. Electron microscopy analyses revealed organelle stress in IECs and administration of the µ-opioid receptor antagonist DALDA inhibited the organelle stress and reduced apoptosis in glafenine-exposed fish [25]. Immune cell recruitment was not examined in this model.

### Lipopolysaccharide (LPS)

LPS, a predominant component of the outer cell wall in gram-negative bacteria, has been shown to elicit pro-inflammatory gene expression in the larval zebrafish intestine. Zebrafish bathed in LPS displayed increased expression of *tnfa*, elevated neutrophil recruitment and this induction was dependent on the adaptor protein Myd88 and Tnf receptor 1 (Tnfr1) [26]. Because fish are fairly resistant to LPS, likely due to the frequency with which they are exposed to gram-negative bacteria in their water, its use for induction of intestinal inflammation presents difficulties [27].

## Environmental induction of intestinal inflammation

Environmental factors have been implicated in IBD onset. Cigarette smoking, for example, is known to lead to an increased risk for Crohn’s disease yet appears to be protective against ulcerative colitis [1]. Stress, infection and NSAIDs have also been implicated in disease pathogenesis, though a specific mechanism for any of these factors has not yet been established [1]. Nevertheless, zebrafish are highly amenable to chemical screens and may prove a valuable model for identifying environmental factors contributing to IBD onset.

### High cholesterol diet (HCD)

In both larval and adult fish fed an HCD, Progatzky and colleagues document an acute and robust recruitment of immune cells to the intestine after feeding [28••]. This recruitment is tightly linked with microbial colonization of the intestine and depends on uptake of cholesterol through NPC1L1 and NFkB signaling. It is interesting to note, however, that although knockdown of *il1b* prevents recruitment of immune cells after an HCD, *il1b* levels are not upregulated in response to the HCD. A prolonged HCD resulted in profound changes, including steatosis, cholesterol deposits in the caudal vein and perturbed intestinal peristalsis [28••]. Newly developed inflammation-responsive transgenic lines (discussed below) may facilitate further research into HCD-induced intestinal inflammation.

### Depleted uranium

Adult zebrafish reared in water containing depleted uranium, a byproduct of nuclear enrichment, resulted in accumulation of uranium in the nucleus and a decrease in the number of calcium-containing mitochondrial matrix granules in IECs [29]. Analysis of the zebrafish intestine revealed disruptions to mucosal morphology and an increase in vacuoles in IECs at the tips of intestinal folds [29]. Changes in bacterial colonization were noted in the depleted uranium-treated groups, though future studies to characterize these changes are necessary. Pro-inflammatory gene expression, immune cell recruitment and barrier function were not assayed in this study. While more basic research is needed, the release of significant amounts of depleted uranium into the environment due to recent military conflicts may be a factor contributing to IBD in war veterans [30, 31].

### Soybean-based diet

The type of feed used for zebrafish can have a strong impact on intestinal physiology and function. Previous studies in other fish species revealed defects in intestinal morphology and increased immune recruitment after they were fed a diet containing soybean meal. When larval fish were fed a soybean meal-based diet, Hedrera and colleagues observed an intestinal neutrophil recruitment phenotype and an increase in Il-1b and Il-8 [32]. Defects in intestinal morphology were not observed, which the authors attributed to the short time frame used for feeding and observation [32].

## Genetic models of inflammation

### Epigenetic regulation of inflammation

A recent paper from our lab has demonstrated that two conserved regulators of DNA methylation are critical for prevention of intestinal inflammation. Loss of function of either *uhrf1* or *dnmt1*, which work as a complex to methylate CpG sites during DNA replication, results in the spontaneous development of intestinal inflammation with perturbed IEC morphology, cell shedding and apoptosis. An in-depth analysis of *uhrf1* mutants also revealed impaired barrier function, increased expression of pro-inflammatory markers in IECs and intestinal immune cell recruitment [33••]. To assay inflammation in vivo, we generated a novel inflammation-responsive transgenic line *TgBAC(tnfa:GFP*) (described below). *uhrf1^pd1092^;TgBAC(tnfa:GFP*) larvae presented with a marked and specific upregulation of *tnfa* in IECs, which was mirrored by an increase in NFκB activation, a downstream component of Tnfa signaling. Interestingly, *tnfa* upregulation was partly microbe-dependent. Knockdown of *tnfa* reduced inflammation, restored IEC morphology, IEC integrity and barrier function in *uhrf1* mutants [33••]. Strikingly, bisulfite sequencing revealed a loss of *tnfa* promoter methylation and suggests that epigenetic control of pro-inflammatory cytokine production may be a contributing factor to inflammatory diseases like IBD [33••, 34•]. This new IBD model illustrates how genetics, epigenetics and environmental factors contribute to the development of intestinal inflammation.

## Infection-based models of inflammation

The study of how bacterial infections in the zebrafish intestine contribute to inflammation is highly relevant to IBD, as loss of microbial homeostasis and aberrant responses to commensal bacteria in the intestine have been implicated in IBD onset [1]. Important for the study of IBD, gnotobiotic techniques have been developed to derive zebrafish under germ-free conditions, which allows one to study the contribution of microbial colonization and specific intestinal pathogens to intestinal phenotypes [35–38]. Zebrafish infection can be performed in larvae or adults, in control or germ-free backgrounds and with or without inflammatory sensitization.

### Salmonella enterica

Immersion of zebrafish in media containing the enteropathogen *S. enterica*, a pathogen known to cause intestinal infection in humans, was shown to predominantly affect the posterior intestinal segment (segment III) and elicit a neutrophil recruitment response to the area [39]. Loss of function of dual oxidase (duox), a protein involved in the production of reactive oxygen species, resulted in persistent *S. enterica* infection and decreased survival of the larvae [40]. Additionally, morpholino-mediated knockdown of Nod1, a member of the NOD family of proteins implicated in IBD pathogenesis, also contributed to persistent *S. enterica* infection [41]. Infection also resulted in enhanced expression of the heat shock proteins *hspa4a, hspa4b* and *hsp70*; enhanced expression of HSP70 has been observed in the intestine of IBD patients and is thought to play a cytoprotective role [21].

### Edwardsiella infection

Infection of germ-free zebrafish larvae by immersion in the strain *Edwardsiella ictaluri* for six hours resulted in increased mortality, reproducible oral and intestinal lesions and occasional penetrations of the intestinal barrier [42]. *E. ictaluri* infection led to a strong induction of both *il1b* and *tnfa* in whole larvae, though intestinal-specific expression and recruitment of immune cells were not examined.

Infection of adult zebrafish with *Edwardsiella tarda*, a known cause of human enteritis in immunocompromised individuals, was pathogenic only after intestinal inflammation was induced by rectal administration of oxazolone and ethanol [43]. *E. tarda* infection in fish with oxazolone-induced intestinal inflammation led to an upregulation of *il1b*, *tnfa* and myeloperoxidase, a gene highly expressed in neutrophils [43].

## Technological advances for the study of intestinal inflammation in zebrafish

Several recent technological innovations have occurred in zebrafish that propel this system to the forefront of intestinal biology and mucosal homeostasis research (Figure 1). Two inflammation-responsive transgenic lines, *TgBAC(tnfa:GFP)* and *Tg(NFκB:EGFP)*, have been shown to report intestinal inflammation, in intact living larvae [33••, 44]. Detection of pro-inflammatory signaling by TNF and its downstream components such as NFκB has been challenging in other systems and often relies on immunohistochemistry or RT-PCR, which precludes real-time analysis of inflammatory events. To further enhance analyses of intestinal inflammation in vivo, several transgenic lines have been developed to monitor immune cell recruitment dynamics. Combining the *Tg(lysC:dsRed)* and *Tg(mpx:EGFP)* transgenic lines, which highlight the neutrophil compartment [39, 45, 46], or *Tg(mpeg1:mCherry)* and *Tg(fms:mCherry*) transgenic lines, which label macrophages [47, 48], with the inflammation-responsive reporters now allows researchers to examine immune cell migration dynamics, response to and production of pro-inflammatory signals in an intact single animal over time. This type of information has not been previously available in other models of IBD due to lack of transparency and the invasive nature of imaging that would be required. Re-analysis of previously published genetic models of disrupted intestinal morphology (e.g. *titania^s450^* and *flotte lotte* [49–51]) in the *Tg(tnfa:GFP)* and *Tg(NFkB:EGFP)* backgrounds or immune cell backgrounds will provide further insight into the onset of IEC integrity defects. Of particular interest is the *titania^s450^* mutant, which displays an increase in IEC autophagosomes [49], as autophagy has been implicated in IBD onset [52].

Our lab has also generated several intestinal-specific transgenic lines that facilitate the analysis of IBD and other intestinal phenotypes. For example, *TgBAC(cld15la-GFP)* is a BAC transgenic line that is exclusively expressed in the intestine beginning at 3 dpf and labels the basolateral surfaces of IECs [53]. *TgBAC(anxa2b-RFP)* is another BAC transgenic in which the apical surface of the IECs is marked, beginning at 4 dpf [33••]. These transgenic lines provide a way to monitor how different inflammatory conditions (genetic or chemical) affect the intestinal epithelium and are also useful for cell sorting experiments to detect the pro-inflammatory gene expression profile specifically within IECs [33••].

Microgavage [17•], which uses a capillary needle to deliver substrates directly to the intestinal bulb, has facilitated several studies of intestinal physiology [33••, 54]. Because immersion approaches can lead to extraintestinal consequences [12], studies examining the effect of a chemical on the intestinal epithelium can now utilize microgavage to determine the acute effects on intestinal morphology, immune recruitment and inflammatory signaling. Repeated gavage experiments can be performed to determine how chronic exposure affects the epithelium over time. Additionally, new microscopy techniques such as selective plane illumination microscopy (SPIM) allow for long-term imaging of the gut, even during peristalsis (unpublished observation, Bagnat lab) [55]. *TgBAC(pllp-GFP)*, a new transgenic line that displays enriched expression in the posterior midgut (segment II) of the zebrafish, highlights endosomal compartments in these highly absorptive cells and allows for live imaging of substrate uptake from the intestinal lumen [54]. Use of this line coupled with co-gavage of epithelial irritants and dextran will allow for acute and chronic assessment of how absorption is physiologically affected by various chemical perturbations.

## Conclusions

Zebrafish have emerged as a powerful complement to existing models of intestinal inflammation. The newly developed inflammation-responsive reporter lines will allow for high throughput genetic and chemical screens to identify suppressors and enhancers of inflammation. Zebrafish mutants hold great potential for translational studies; next generation sequencing of human patients for variant alleles of known IBD susceptibility genes can be analyzed in fish mutant for those genes to determine whether or not they are pathogenic. Overall, chemical-based models of intestinal inflammation have proven useful, but are limited as they only fulfill some of the criteria for an IBD model outlined above [12]. In contrast, newly introduced genetic models [33••] offer a comprehensive platform for IBD research that presents unique features not available in other experimental systems. A foundation has been laid such that future studies in zebrafish can begin to forge new territory in characterizing the initial events that occur during disease onset.
